# *If I was more informed about what exactly they do*: perceptions of Botswana district hospital healthcare providers about World Spine Care

**DOI:** 10.1186/s12998-019-0250-2

**Published:** 2019-07-03

**Authors:** Mufudzi Chihambakwe, Laura O’Connor, Penelope M. Orton, Maria A. Hondras

**Affiliations:** 1 Department of Chiropractic, Faculty of Health Sciences, Durban University of Technology, 11 Ritson Road, Durban, 4001 South Africa; 2 Department of Nursing, Faculty of Health Sciences, Durban University of Technology, 11 Ritson Road, Durban, 4001 South Africa; 3 Department of Anesthesiology, School of Medicine, University of Kansas Medical Center, 3901 Rainbow Blvd. Mail Stop 1034, Kansas City, KS 66160 USA; 4 World Spine Care Research Team, 801 North Tustin Avenue, Suite 202, Santa Ana, California, USA

**Keywords:** Botswana, Healthcare providers, Interprofessional relations, Organisational culture, Perceptions, World spine care

## Abstract

**Background:**

In 2011, World Spine Care (WSC) opened their pilot clinic at the Botswana Mahalapye District Hospital (MDH) aiming to develop a low-cost model of evidence-based spine care for underserved communities. Providing sustainable, integrated, evidence-based care will require buy-in from local healthcare providers (HCPs) and the communities served. The purpose of this project was to understand how MDH HCPs perceive WSC.

**Methods:**

We used a qualitative descriptive methodology to conduct individual, semi-structured interviews with MDH HCPs who had some familiarity about WSC services. Interviews were conducted in English, audio-recorded, and transcribed verbatim. We used an iterative coding process for thematic content analysis and interpretations were regularly reviewed by all co-authors.

**Results:**

In March 2017, interviews with 20 HCPs, from diverse disciplines with a range in years’ experience at MDH, revealed three overlapping themes: knowledge about WSC and spinal related disorders, perceived role of WSC, and challenges for WSC integration. Participants who attended WSC conferences or self-referred for care were more informed and, generally, held positive perceptions. Participants lacked knowledge about managing spinal-related disorders, asserted hospital protocols did not meet patient needs, and perceived WSC is ‘filling a gap’ to manage these conditions. There were mixed perceptions about care received as WSC patients; some ultimately obtained relief, while others reported the treatment painful and unfamiliar, discharging themselves from care. Challenges to integrate WSC into the healthcare system were: lack of knowledge about scope of practice and unclear referral pathways; reversing the isolated care WSC provides by increasing collaboration between WSC and hospital staff; and, high turnover of WSC clinicians that undermines program sustainability.

**Conclusions:**

MDH healthcare providers had adequate general knowledge about World Spine Care and spinal-related disorders, but did not understand the WSC scope of practice nor referral pathways to and from providers. Participants advocated for greater collaboration between WSC and hospital staff to increase acceptance and integration to deliver spine care services and foster wider adoption of the WSC model, particularly if WSC expands services across Botswana. Future efforts that incorporate interviews with patients and government officials also can provide valuable perspectives to achieve sustainable, integrated, evidence-based spine care.

**Electronic supplementary material:**

The online version of this article (10.1186/s12998-019-0250-2) contains supplementary material, which is available to authorized users.

## Background

Musculoskeletal healthcare globally is in crisis [1]. Musculoskeletal (MSK) disorders are the leading cause of disability worldwide with under-resourced communities most vulnerable [2]. Furthermore, healthcare systems in developed countries grapple with effective management strategies for MSK disorders, in general, and chronic low back pain in particular [3, 4]. Those most affected are people in low- and middle-income countries who tend to have the least access to primary care [5] let alone musculoskeletal care. The World Spine Care non-governmental organisation (NGO) is attempting to address this challenge.

World Spine Care (WSC) is dedicated to providing evidence-based spine care to underserved communities around the world [6]. The organisation was founded in response to the growing burden of spine-related disorders, particularly in lower- to middle-income countries. The WSC clinical team consists of predominantly chiropractors as well as physiotherapists with specialised training for spinal disorders. In 2012, WSC established their pilot project in the sub-Saharan African country Botswana by opening two clinics in the Central District to provide spine care for patients in the local community [4]. The Mahalapye District Hospital (MDH) houses one of the WSC clinics and the other clinic is located in the nearby village of Shoshong. Armstrong [7] described the preponderance of patients attending WSC clinics in Botswana as female, elderly, of low income and education levels, and often presenting with at least one co-morbid condition.

Sustainability of WSC clinics in Botswana and elsewhere requires “buy-in” from community stakeholders including the local healthcare community. With initial support from the Botswana Ministry of Health for WSC to establish spine care centres in country, integrating the WSC model of care into the existing healthcare system can, in part, address sustainability challenges. Developing effective interprofessional teams, with open communication, patient- and family-centred care, and provider integration are several essential qualities for successful health systems integration [8, 9].

In addition, a relationship exists between how individuals perceive a phenomenon and how they behave towards it. For example, a positive attitude may influence compliant behaviour with a new system [10]. Perceptual disparity ensues when subjective interpretations of two different groups about a given phenomenon are contradictory, often establishing friction between groups [11]. The organisational culture of the MDH may impact the way healthcare providers interact with and perceive WSC. Similarly, the WSC organisational culture and leadership may influence health system integration. The culture can significantly impact the levels of commitment and resultant attitudes and behaviour of the individuals within those organisations [12].

There are a number of factors that affect perception and these factors may influence the manner in which MDH healthcare providers (HCPs) interact with WSC staff. Perception and attitudes affect behaviour [13] and attitudes can change depending on the level of knowledge available. One of the aims of WSC is to provide care that is culturally relevant and locally adapted [4]. Thus, a greater understanding of MDH HCP perceptions of the WSC program is important to meet this aim and foster sustainability. The purpose of this qualitative research was to explore healthcare provider knowledge, attitudes and perceptions about the WSC program at a district hospital in Botswana.

## Methods

We conducted a descriptive study of MDH healthcare provider perceptions about the World Spine Care program. A descriptive qualitative methodology [14] was used, situating the lead author at the MDH to gather data and subsequently engaging co-authors in the interpretive process to help make sense of the data [15].

### Setting

**Fig. 1:**
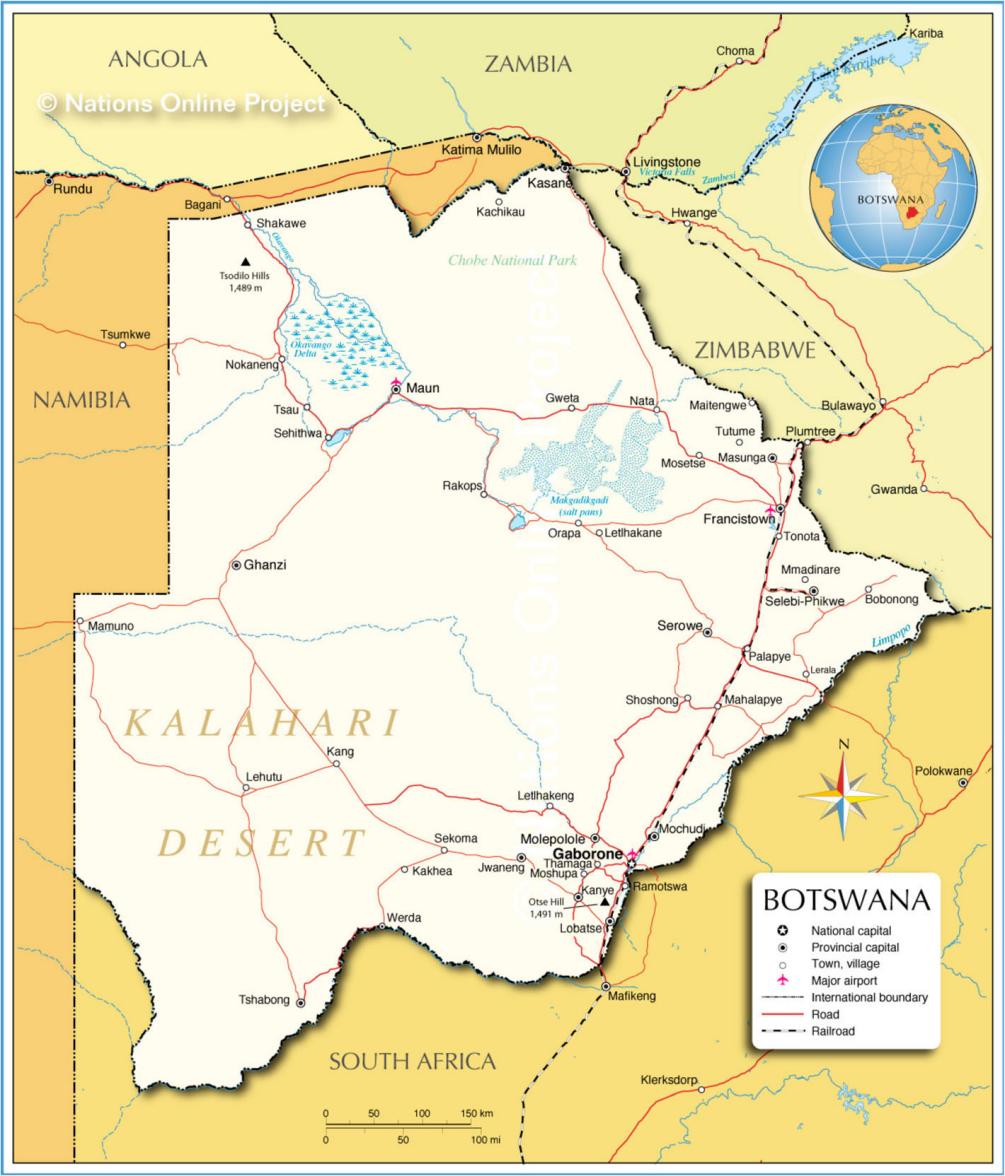
Map of Botswana (Permission from Editor Klaus Kästle, One World –© Nations Online, OWNO, nationsonline.org on 20 September 2014.)s

This study was conducted in Mahalapye, Botswana, where the population is approximately 41,000 [16]. Mahalapye is located in the Central District nearly 200 km northeast of Gaborone (Fig. 1) along a major highway connecting the country’s two largest cities, Gaborone and Francistown. The MDH is found on a turn off from the highway along a tarred road without paved shoulders. Vendors set up their stalls across the road from the hospital entrance, preparing meals and snacks during the workday where many of the healthcare providers frequent.

**Fig. 2:**
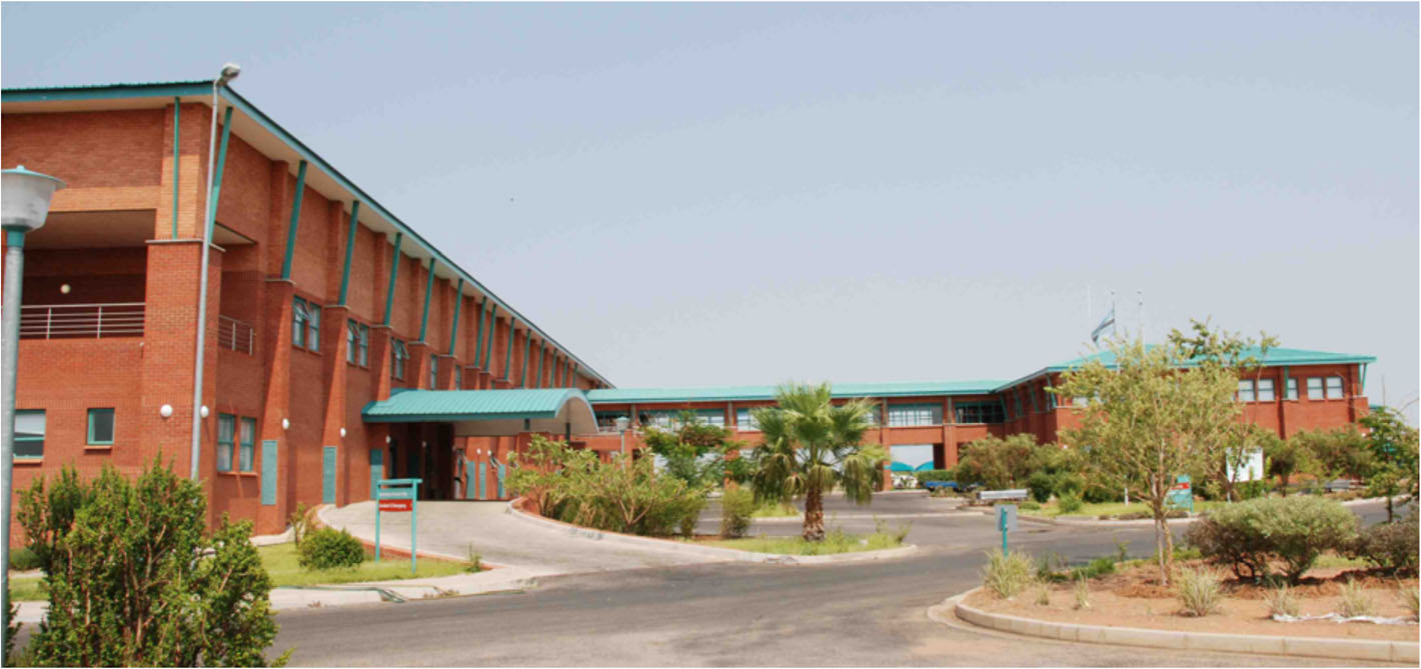
Mahalapye District Hospital (Photo permission from Dr. Richard Brown via email on 24 October 2018)

The superstructure of the hospital rises starkly against the surrounding areas of sparsely vegetated dry plains. The hospital is a large, red brick structure with teal green finishes (Fig. 2), blending well with the national Botswana colours of sky blue, white and black. The early mornings are a rush, with dayshift healthcare employees arriving in various forms of transport: some in cars, others by public transport, and many employees walk to the hospital. Patients can be seen sitting patiently on benches within the hospital early in the day, including elderly women, aged men slowly shuffling by and having hushed conversations, and younger patients who are either mothers with children or trauma cases waiting to be attended. The hospital tends to quieten down in some departments by mid-day, while other departments always seem to have patients waiting. The MDH sits as a hub of activity in the town.

World Spine Care has two clinics in the Botswana Central District: one in the village of Shoshong, 40 km west of Mahalapye, and the other located in the MDH outpatient physiotherapy ward. WSC volunteer clinicians alternate days of the week between Shoshong and Mahalapye.

### Participants

The sample for this study was obtained from healthcare providers working at MDH at the time of enquiry (*n* = 430), including medical officers, physiotherapists, surgeons and nurses (to name a few) and excluding drivers and outsourced staff. Participants for this study had to be English-speaking, employed at MDH, and had to have some familiarity with World Spine Care.

Participants were recruited by purposive and snowball sampling, wherein MC visited the MDH several months before data collection to identify potential participants based on various roles in the hospital and their engagement with WSC. When data collection was underway, initial participants suggested additional HCPs to approach for participation. The sample size was not predetermined. Rather, during two weeks of fieldwork, MC strived to conduct interviews until reaching data saturation. MC transcribed and began the analytic process after each interview; thus he was able to assess the point where no new themes or ideas were generated from interviews [17].

### Data collection

Semi-structured interviews were conducted in participant workspaces allowing them to be relaxed and comfortable, in a familiar setting, when responding to research questions. The lead author (MC) began each interview by asking a ‘grand tour’ question: *Can you tell me your thoughts about World Spine Care and its place in the Botswana healthcare system?* The researcher used prompts for participant responses that did not address the main research problem [18]. Prompts to gather information for participant knowledge, attitudes and perceptions included:

- Knowledge prompts
  - What do you know about WSC’s model of healthcare?
  - What do you know of the WSC activities?
  - What is your understanding of WSC’s goal at the MDH?
- Attitude/perception prompts
  - What has been your experience of WSC?
  - What is your opinion of the WSC?
  - How can WSC improve their service?

Interviews were captured electronically with a digital voice recorder and transcribed verbatim into a text document for analysis. After most interviews and daily during data collection, MC made journal entries reflecting on the interviews and study process. Basic demographic data were collected to describe the study sample, including: age, gender, occupation, length of service at MDH and department of employment at the hospital.

### Data analysis

Data were analysed using conventional content analysis [19] whereby MC read each transcript to get a sense of the data and identify key text within each transcript that covered areas of interest. The data were then interpreted and manually condensed into meaning units, codes, categories and themes, as described by Graneheim and Lundman [20]. Data were grouped to form content areas that allowed horizontal analysis across all interviews. Key words and phrases were identified from the content areas to make up the codes [21]. Codes were further refined and placed in categories. Ultimately each category was analysed in context with all transcripts to generate sub-themes and themes. MC subsequently prepared his interpretations of the data [14], which were regularly reviewed with co-authors to ensure well-reasoned explanations. Some deductive reasoning also was used by constantly checking emerging themes against the data [22].

### Researcher team profiles

Given that personal and professional interests will inevitably shape interpretations [23, 24], we acknowledge author experiences for the analytic process. MC is an African male, in his mid-20s, raised in Zimbabwe. This project formed his master’s thesis in chiropractic, where he had two-year’s clinical experience in outpatient clinics in Durban, South Africa. LO is white, in her early 40s, residing in KwaZulu-Natal, South Africa. She is a chiropractor who has worked in South Africa and Ireland and currently holds a senior lecturer position in the Durban chiropractic programme. She has mentored many chiropractic students to obtain their master’s in chiropractic, covering both quantitative and qualitative research approaches. PMO was born in Zambia and raised in Zimbabwe where she completed her general nurse training with additional nurse training and education in the United Kingdom and South Africa. She has 40 plus years working as a professional nurse in several countries. She has been in academic occupational health nursing for 13 years; has supervised and examined numerous master’s-level projects in South Africa; and, her research interests are in specialist nurse education particularly occupational health nursing. MAH is a white, Greek-American in her early 60s, born and raised in the eastern United States. She is a chiropractor with more than 30 years’ experience as a manual therapist and clinical research academic and 12 years’ experience with qualitative research efforts. She is a WSC research team volunteer, has conducted ethnographic research in the Botswana Central District, and has mentored or examined numerous master’s level projects in the US, Botswana and South Africa.

### Data quality

To increase project trustworthiness, we describe three qualitative practices: credibility, confirmability and transferability [25]. We aimed for credibility and confirmability in several ways. First, earlier visits by MC and MAH provided an appreciation for the cultural context of healthcare delivery in general and musculoskeletal care in particular. Both authors participated in the 2016 WSC conference in Mahalapye and visited WSC clinics in Shoshong and MDH, interacting with villagers, local HCPs, and WSC volunteers. MAH also conducted research among Shoshong villagers to examine the burden of living with and caring for people who live with MSK conditions [23, 26, 27]. Second, we provide explicit details for the methods used to interview, transcribe and analyse data for the current project. Third, MC regularly reviewed and discussed his analysis and interpretations with co-authors. We aimed for transferability by providing contextual information about the fieldwork site and participant professional roles, allowing readers to discern applicability to: their own setting, other settings in Botswana, and other WSC clinic locations.

### Ethics

Four committees granted permission to conduct this study: Health Research Development Committee of the Botswana Ministry of Health, Mahalapye District Hospital Health Research and Development Division, Durban University of Technology Institutional Research Ethics Committee, and World Spine Care Research Committee. Written consent was obtained from participants before each interview. Anonymity was safeguarded by excluding the name, age and specific discipline of participants in reports of this work; rather, participant identifiers with direct quotes include the letter P plus transcript number and broad-based professional role in hospital.

## Results

Findings are based on 20 in-person, semi-structured interviews with MDH healthcare providers (7 women and 13 men). Interviews averaged fifteen minutes and were conducted in March 2017. The HCP length of service at MDH included: 4 who had been in service for less than one year, 6 with one to five years’ service, 6 with more than five but less than ten years, and 4 with ten or more years of service. HCP countries of origin included Botswana, Zimbabwe and the Democratic Republic of the Congo. Disciplines were diverse and are displayed in Table 1.

**Table 1:** Roles and disciplines in hospital for 20 participants

Professional role description with direct quotes	Disciplines represented
Allied health	Clinical psychologist; dental assistant; healthcare auxiliary; orthotist; pharmacist; physiotherapist; radiographer; surgical orderly
Medical officer	Dentist, family physician (2); hospital administrator with medical licensure (2); medical officer (2); orthopaedic surgeon
Nurse	Nurse (4)

**Fig. 3:**
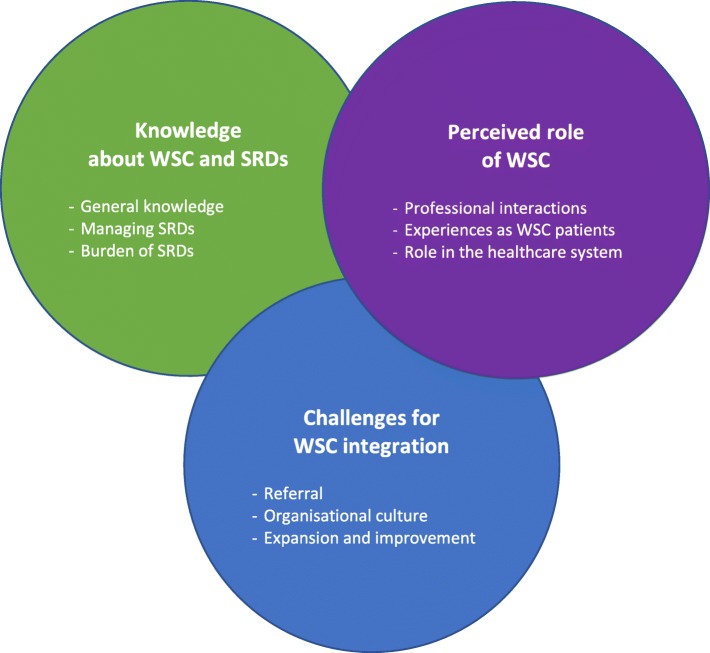
Themes and sub-themes from semi-structured interviews with 20 healthcare providers at the Mahalapye District Hospital (Abbreviations: SRDs = spinal-related disorders; WSC=World Spine Care)

There were three overarching themes from interviews: knowledge of WSC and spinal-related disorders (SRDs), perceived role of WSC, and challenges for WSC integration. Each theme included several sub-themes with overlapping contextual perceptions and experiences (Fig. 3). We include illustrative quotes for themes and sub-themes next, with additional exemplary quotes presented in Additional file 1.

### Knowledge about WSC and SRDs

#### General knowledge

All HCPs interviewed had heard of WSC; most knew WSC also had a clinic in Shoshong; however, few recognised that WSC had clinics in other parts of the world. Participants who had interacted with WSC either professionally or as a patient described what they had seen and experienced. They spoke about: WSC using manual therapies to treat SRDs; community projects and exercise programs conducted by WSC [Additional file 1]; and, educational conferences or workshops WSC hosted. One participant mentioned the beneficial role WSC serves in hospital, noting:

*…about the chiropractors … with regards to what I have encountered in primary care, you know musculoskeletal pains and such kinds of things, they (WSC) can be highly of help [P3, Medical officer]*

Other participants felt their understanding of WSC was superficial or even inadequate and said they would not be sure when to refer patients to WSC providers. Some mentioned they did not know the WSC scope of practice nor daily operations.

*The problem is we don’t know much about … the department. Or the services that are provided… [P11, Medical officer]*

*If I was more informed about what exactly they do, what their specialty entails, so that when I spot their client somewhere in the wrong environment I can get it to them. You see what I mean? I don’t know much about them. [P7, Allied health]*

Participants who had interacted with WSC described the system of patient care as efficient though different, WSC workshops and conferences as informative, and interactions with WSC clinicians as pleasant. Participants who had not interacted with WSC clinicians expressed a desire to interact and blamed the lack of interaction on time constraints and heavy workloads. In contrast, some participants felt that WSC clinicians worked in isolation and were not forthcoming about WSC operations as these HCPs knew about WSC but did not know enough to interact with them.

Several participants revealed ideas they have about the causes of spinal-related disorders and provided context for understanding the role of WSC. The nature and prevention of SRDs was a common thread:

*I would say Africans are not really well educated about spine care, how to care for their spines … the type of exercise they do, maybe the type of job they do, somebody will just lift something heavy without caring for the spine. [P10, Nurse]*

Another participant added a strand of disregard to the lack of understanding:

*There are so many things that we don’t understand. Well actually we understand them but we ignore them. [P12, Medical officer]*

In general, HCPs perceived a need for better prevention and care for SRDs and the potential seriousness these disorders have on people’s everyday lives.

#### Managing SRDs

As the HCPs described what they knew about WSC, many stated they lacked knowledge of SRDs to adequately manage them. Participants spoke about SRDs ranging from spinal cord trauma to backache. However, regardless of severity, participants felt ill-equipped to care for disorders of the spine:

*Honest truth is … I don’t think many of us know as much as we need to know about lower back pain. Because you find that for most of the time you don’t really pay attention to the first two words, lower back – it’s just pain. And you just prescribe analgesia and go. Which is not really the proper management for lower back pain, you know? [P19, Medical officer]*

Other participants described SRDs as something to prevent rather than treat. One HCP asserted the nature of SRDs in foreboding terms:

*Even the wellness. Because when you talk of spine care, akere (you see) you are not saying people…. People should come to you when you are sick? People should be able to take care of their backs because at the end of the day… it plays a very pivotal role. Once you are down with that low back ache, you are doomed. [P11, Medical officer]*

Another participant described how his perspective about managing SRDs changed after attending a presentation by WSC staff:

*We usually request x-rays from each and every patient complaining of this. But when you sit down and look, that x-ray is not even going to change how you thought of your management. We have been spending a lot of money on the cases where we see that we could have done with less resources and less costs. [P4, Allied health]*

Thus, WSC presence in hospital may challenge the status quo for SRD management to more closely align with evidence-based principles.

#### Burden of SRDs

A common perception among the HCPs was that SRDs are considered ‘a norm’ amongst Batswana (Botswana natives)*,* because of the rural lifestyle many have in country. One participant said:

*We do get a lot of lower back pains. That’s what we often see in our consultations, especially in the elderly because of all the manual labour. You know Batswana tend to use a lot of manual labour to get by. So, lower back pain is quite a significant problem in our population. [P19, Medical officer]*

Another participant added to the cultural context for SRD burden and identified paths to consider for education and care:

*Spinal pain is very common, especially to us Africans and us Batswana. So that’s the most complaint that people come with alone. By nature, our duty as Batswana, we do more laborious work. We put more strain on our backs, we don’t know how to carry our spine so I think that’s the entry point as to how can we prevent the lower back pain and moving forward what can we do if we experience symptoms of the lower back pain. [P4, Allied health]*

Several HCPs considered WSC a potential resource to manage SRDs that are pervasive amongst MDH employees from routine hospital duties.

*How to lift, akere (you see?) … we work in a hospital setting where people will be lifting boxes, patients collapsing and what have you. They should be able to train people on those parts of ... because I talk of wellness … wellness you are talking prevention, isn’t it? ... a lot of people here are suffering from back problems. [P11, Medical officer]*

### Perceived role of WSC

#### Professional interactions

There were mixed attitudes towards the role of WSC at MDH. Some participants felt WSC was doing relevant work in hospital, based on personal interactions, while others sensed WSC was not doing enough to promote their services. One participant considered limited interprofessional collaboration the result of WSC ‘operating in isolation.’

*They should continuously inform the incoming officers that there is this department. According to me … in this hospital you (WSC) are functioning in isolation [P11, Medical officer]*

Others had attended workshops and conferences WSC hosted and found them informative:

*Yeah, I did attend the WSC conference in Cresta (venue). Yes, last year. I learned a lot of things there. Of course, from the clinical perspective. [P4, Allied health]*

#### Experiences as WSC patients

HCPs who also were patients at the WSC clinic described how their interactions as patients influenced their perceptions. One participant offered she would not have interacted with WSC had she not been suffering from back pain:

*I heard about it when I was suffering… pain … on the lower back. The doctors used to give me analgesics but with no improvement. So, I heard about those people, the spine care program. I referred myself to them. The procedure was very painful. But the effect is very good … after, after that exercise. So, I went there about maybe six times ... after that I discharged myself. [P14, Nurse]*

The potential disparity between patient and provider expectations is illuminated in the expanded version of this participant’s comment [Additional file 1], where she provides a vivid description of self-referral; the diagnostic process, treatment endured, and relief obtained at the hands of WSC providers; and, declining subsequent follow-up.

Several participants admitted negative perceptions resulting from the unexpected nature of their own treatment experience or what they heard from other patients:

*Because when I went there my expectation is to get better. But when I don’t get better and no one is telling me as to why I am getting the symptoms which are worsening. [P4, Allied health]*

In contrast, despite initial discomfort, one participant noted how her condition improved after returning for treatment:

*I was scared because I knew I was going to experience that pain. But at the end of the tunnel, I am going to better. So that’s what happened to me. But since then I am much, much better. [P16, Nurse]*

Hence HCP perceptions of WSC were strongly influenced by their experiences as patients.

#### Role in the healthcare system

Participants considered WSC services complementary to existing hospital services.

*I think its complementary to other departments which deal with any issues that have to do with spinal problems … spinal rehabilitation … ah, I think it’s a very good option other than physiotherapy. [P7, Allied health]*

One participant sensed that, while the treatment methods were not the same, WSC was reducing the workload for MDH physiotherapists:

*Actually, they assist for us, so that we can share our workload with them. So, I think WSC is doing uh ... its beneficial you know. It has been supporting our patients, so I don’t find any problem with them. [P2, Allied health]*

Thus, participants perceived the role of WSC in the realm of musculoskeletal health with a particular focus on spinal health.

### Challenges for WSC integration

#### Referral

For the most part, HCPs were willing to refer patients to the WSC clinic; however, the referral pathway to WSC was unclear. One participant said:

*So, to me there was still no proper channels of referral…sometimes when you knock there, there is no one. Sometimes they do come here and they look for someone but there is no one to speak to. So… we need a system of referral. [P4, Allied health]*

This lack of clarity for the referral process was a matter of concern, where the respondent continued, suggesting some patients were not adequately cared for:

*I think that is why maybe you are losing a lot of patients. Sometimes patients they do present themselves to the WSC and there is a need to refer those patients to the new medical officers or the surgeons but we still doing it haphazardly. [P4, Allied health]*

Other HCPs, who typically treat musculoskeletal conditions conservatively such as physiotherapy and occupational therapy, also noted confusion with the referral system:

*Some confuse (us) because they don’t have the in-depth (knowledge) of some other professions … for example there is big confusion between prosthetics and orthotics, occupational therapy, physiotherapy. You find somebody just writes (an unclear referral note) ... he knows something is there but he doesn’t know really what their parameters are. [P6, Allied health]*

Consequently, unclear referral pathways contribute to HCP attitudes toward WSC services.

#### Organisational culture

Several HCPs expressed uncertainty regarding the future of WSC at MDH or in what capacity the WSC exists in hospital:

*…don’t know how long they will stay as World Spine Care, but I know they are training people to take over the clinic. [P1, Medical officer]*

*So, I don’t know ... whether they are private sector … or they are part of the main hospital setup? … like now, if I want to go there I will not know whether to go through somebody else or go straight to them. [P11, Medical officer]*

Participants mentioned time constraints, long work hours and the limited labour pool often constrain their ability to interact with other hospital staff:

*I have a high volume of patients so for me it’s difficult to communicate to others very frequently. [P9, Allied health]*

*Because of so many patients coming in…you can’t pause. You can’t even pause to talk to each other, you know. [P7, Allied health]*

Constraints are further compounded by high staff turnover where a number of HCPs serve only a few months’ tenure in hospital before being transferred to other hospitals or because certain positions are not filled for various periods of time. One participant noted:

*The rehabilitation team includes physiotherapists, orthotics, occupational therapists but occupational therapists right now we don’t have any. They are all transferred. So, they are not there. [P2, Allied health]*

In combination, these factors limit or even strain interprofessional communication amongst MDH HCPs and departments. This ultimately may impact the knowledge and perceptions HCPs have about WSC personnel and programs.

#### Expansion and improvement

There was consensus among participants that WSC should expand spine care services to other centres in country.

*I think they have got some preliminary results that show that indeed this thing is needed in Botswana and we have seen how a lot of people, they go untreated for a lot of years up until they die. But it’s time now it’s spread to other areas where people are still suffering. [P4, Allied health]*

Participants indicated, for this to happen, WSC would need to increase staffing, gain greater government support, and improve integration with local healthcare professions. Two participants made specific suggestions to improve spine care services:

*I think maybe if they can train even some nurses there. Nurses are the core of the health system. Because these are the people who are always there seated with patients. [P4, Allied health]*

*Not only health-based care but community-based care, because we are moving now in terms of primary care to be more community-based than institutional based. [P3, Medical officer]*

Participants voiced concern about the turnover rate of WSC clinicians that undermines sustainability of the program and limits organisational aims in country:

*I think the issue of volunteers coming in and going, I think it’s going to disturb the program in a way moving forward. Because as Motswana we normally establish that rapport with the people we first see. We need continuity. [P4, Allied health]*

Many participants made practical suggestions for WSC to improve and expand their reach. Recommendations included: greater use of media to advertise spine care services, continuing education of healthcare providers within the hospital, and expanding patient education about the nature of care they may receive.

## Discussion

This descriptive qualitative study explored the knowledge, attitudes and perceptions of Mahalapye District Hospital healthcare providers about World Spine Care. Perceptions that emerged were generally positive and influenced by a number of factors. The perceivers included MDH HCPs from different disciplines, countries and cultures. The manner in which HCPs interacted with WSC clinicians in hospital as well as during workshops conducted by the NGO’s leadership and research team influenced perceptions. MDH organisational culture within Botswana’s healthcare environment, along with the global and local burden of spinal-related disorders, provided context that also shaped perceptions.

The knowledge participants had about the WSC program varied with the type of exposure. Unsurprisingly, those who had attended workshops or had been to the WSC clinic for treatment were more informed about WSC and, generally, had more positive perceptions. HCP discussions about the burden and management of spinal-related disorders highlighted shortcomings in their setting and proposed WSC is ‘filling a gap.’ Participants asserted current protocols to manage SRDs in hospital were not meeting the needs of their patients. These sentiments concur with those of Abou-Raya and Abou-Raya [28] who lamented the lack of emphasis on musculoskeletal education in medical school curricula as well as rudimentary MSK diagnostic acumen in clinical settings.

Moreover, participants expressed frustration about their experiences treating pain, in general, and the elderly, in particular. These findings are consistent with other district hospitals in Botswana where local healthcare workers struggle to assess and care for pain patients given the paucity of effective diagnostic procedures and treatment protocols [29]. Consequently, many participants welcomed the WSC approach to manage pain and SRD sequelae acknowledging the WSC model challenges the status quo to manage these conditions at the MDH.

While HCPs conceded the WSC approach may be beneficial and sorely needed for pain care, a number of participants stated they were not familiar with chiropractic. This is relevant because, although WSC does not promote itself as a chiropractic organisation [4], most WSC volunteers are chiropractors and the lack of knowledge about this discipline could negatively impact organisational relations. This amplifies the need for continued interprofessional education about spine care as well as chiropractic care.

A variety of insights about the perceived role of WSC emerged from HCP experiences. Most participants were open to engaging with WSC but cited time constraints as a barrier. Several participants had attended the WSC clinic as patients, with a mix of positive and critical comments about the care received. Some ultimately obtained relief, while others reported the treatment painful and unfamiliar and had discharged themselves from care.

The biggest challenge participants identified to integrate WSC in the healthcare system was lack of knowledge about the scope of practice and, thus, referral pathway(s). Several participants stated that while they had heard about WSC they did not know enough about WSC to refer their patients to them. This is consistent with other studies [30, 31] that found while physicians are willing to refer patients to chiropractors, few will initiate a formal communication with a chiropractor because of a lack of knowledge and experience with chiropractic. This means that patients directly contact the chiropractor which poses challenges for continuity and quality of care. This may occur because physicians do not have a protocol to refer to chiropractors or there may be interprofessional bias [30]. It is unlikely interprofessional bias influences our findings because none of the HCPs were opposed or resistant to WSC in hospital and many ended interviews recommending WSC expand to other areas of the country.

One way the role of chiropractors in a multidisciplinary setting has evolved is by filling the role of primary spine care provider (PSCP), a ‘general practitioner’ for spinal disorders [32]. The PSCP is described as a healthcare professional well able to consider the nuances of spinal disorders and use evidence-based practices, knowing when and to whom to refer for specialist management [33]. Paskowski et al. [34] found the primary spine care model led to a decrease in the burden of spinal disorders and more efficient healthcare spending at a Massachusetts hospital. Successful implementation at MDH will require: support from hospital administrators and government entities who oversee operations; hospital-wide education about the basic tenets of effective, evidence-based musculoskeletal care; and, engaging the larger community with educational drives about spinal health and how the primary spine care practitioner is an ideal entrée for spinal related disorders.

Another challenge the WSC NGO may face is sacrificing isolated care for initiatives that integrate care with nurses. While there is some literature to suggest Motswana nurses are overworked, bear the weight of implementing new initiatives, and have little time to rest [35], integrating nurses into spine care services may be valuable for wider adoption and acceptance of the WSC model. One participant described ‘*nurses as the core of the healthcare system.’* Others also have described nurses as the cornerstone of healthcare settings [36] and an integral part of community-based interventions.

Further on, participants described a lack of interprofessional interaction amongst different departments in the MDH. Specialized HCPs and allied health providers described little interaction with other HCPs in hospital and the chance of interacting with WSC clinicians was slim. In a systematic review of qualitative studies in primary care [37], the lack of clearly defined roles and competencies, along with the lack of interprofessional training were barriers to develop collaborative professional practices that improve the quality of patient care. On the other hand, Salsbury and colleagues [9] found that teamwork and an openness to feedback can enhance the ability of chiropractors to work in interdisciplinary settings. Thus, overcoming essential barriers to effective interprofessional collaboration by clearly defining the roles and competencies of healthcare team members [9, 37], engaging in team building and interprofessional training activities [9, 37], and exploiting existing networks with formal and informal linkages among healthcare providers [8] can improve organisational commitment among HCPs [12] and foster “buy-in” from other community stakeholders to sustain spine care services [8].

This study is not without limitations. Because there was limited time for fieldwork as part of a master’s thesis and because HCPs had busy schedules, the number and duration of interviews was restricted and transcripts were not returned to participants for comments. However, data saturation was reached after 15 interviews, an additional five interviews were conducted to ensure redundancy [38], and participant contributions and insights were valuable for stakeholders to consider. Funding was not available for interpreters and translators, so interviews were conducted in English. While English is the official language in country and in hospital, the researcher sensed hesitancy in responses from several participants who may have been more comfortable in their vernacular language. At the time of this project, MC planned to volunteer as a WSC clinician upon completion of his studies; this may have influenced interpretations of the data.

World Spine Care continues work in Botswana and it is unclear how interprofessional dynamics may change when Motswana clinicians lead WSC clinic operations. Similar studies can be conducted at WSC sites in other countries to compare findings and inform NGO development. Likewise, investigating the perceptions of lay patients as well as government officials about the WSC NGO also will be valuable.

## Conclusion

This qualitative study explored the knowledge, attitudes and perceptions healthcare providers have about the World Spine Care NGO at the Mahalapye District Hospital. HCPs had adequate general knowledge but did not understand the WSC scope of practice nor referral pathways to and from WSC providers. HCPs thought WSC operated in isolation and encouraged interaction with hospital staff to improve care for patients with spinal-related disorders. HCPs who had been patients at the WSC clinic experienced discomfort and uncertainty about treatment procedures, but several ultimately received relief from their chief complaints. HCPs urged WSC to include nurses to deliver spine care when establishing clinics in other parts of Botswana. Studies can be conducted at other sites where the WSC NGO establishes services to examine similarities and differences with our findings. Incorporating interviews with patients and government officials also can provide valuable perspectives to achieve sustainable, integrated, evidence-based spine care.

## Additional file

Additional file 1: Exemplary quotes for themes and sub-themes. (PDF 80 kb)

## Abbreviations

HCP: Healthcare provider
MDH: Mahalapye District Hospital
MSK: Musculoskeletal
NGO: Non-governmental organisation
P#: Participant number
PSCP: Primary spine care provider
SRDs: Spinal-related disorders
WSC: World Spine Care
