# Supplementary material for: If I was more informed about what exactly they do: perceptions of Botswana district hospital healthcare providers about World Spine Care
Source: Chiropr Man Therap. 2019 Jul 3;27:28. doi: 10.1186/s12998-019-0250-2 (PMC6607584; doi:10.1186/s12998-019-0250-2)
Supplement: Supplementary file 1 — Exemplary quotes for themes and sub-themes. (PDF 80 kb) [file 12998_2019_250_MOESM1_ESM.pdf]

Additional file 1. Exemplary quotes for themes and sub-themes

Knowledge about WSC and SRDs

General knowledge

…but I know they are training people … Like, I think there will be a training; there was a training last Thursday. They showed people how to examine. Then we also had exposure to international people coming here for conferences. There, apart from the spine people, our orthopaedic surgeons –also get exposed to these high-class people that are doing a lot of spine operations and things like that … so it's a lot of learning, upscaling of skills and things involved there. Plus, the community, they have gone to schools, they have encouraged – I mean, they have shown teachers what they should look for in students and so … it's very, very good. [P1, Medical officer]

The problem is we don't know much about … the department. Or the services that are provided. If you were to ask me like, 'what services are they providing?' (shrug) I wouldn't know much. I'd be just talking of no … let them teach us how to … take care of ourselves. [P11, Medical officer]

No, I don't think it's out there yet. Because we had requested … indications, like for referral. What they … they would usually like to see. What they deal with. But I haven't seen it yet. So most of us wouldn't know who they would see, how they would help them. [P18, Allied health]

Managing SRDs

I have heard about it but it's not something which clicks my mind to say, 'It's so serious or whatever.' But, eh, the time my family member got sick it's when it opened my mind. Because we have been going to so many hospitals you know, it's when you open your mind. Otherwise before that it was not much into me. [P15, Allied health]

A lot of ignorance even in the medical fraternity exists. Uh, not just amongst physicians, even amongst those who, you know, have to go to the accident scenes, the paramedics. You will be surprised, many of them don't know what they ought to do, you know, to either prevent or minimize the effects of spinal cord trauma. [P20, Medical officer]

I'd say these are the first people who see the patients, so to me they can diagnose the problems that the patients are presenting with... eh, they can identify those patients. They do these yoga things and what-what. They will be meditating with the patients. They do the massages. I knew that if you complained of the spine the first thing that I thought of was surgery but I just learned that now there are some red flags and the x-ray … are not important as long as they are not going to change anything from your management. You don't have to request a lot of the x-ray … but from the clinical point of view we usually request for the x-rays from each and every patient complaining of this – go and do the x-ray. But when you sit down and look, that x-ray is not even going to change how you thought of your management. I think from that I could pick that a lot of things have not been going well. We have been spending a lot of money on the cases where we see that we could have done with less resources and less costs. [P4, Allied health]

Burden of SRDs

And it's good for us Batswana because we are always (higher pitched voice) you don't have time! Sometimes we are … we neglect ourselves akere (you see), sometimes we still have something. Maybe like I said I fell, maybe when I am having a pain that pain back, I'll say, no it's because I once fell. But when they are here and they have opened up, we are so … free. Eheh … I think that will be, uh … lessen the burden of coming to the hospital like what I am saying. I can say I am no more a … a patient who is trouble to orthopaedic surgeon or whatever. [P16, Nurse]

Perceived role of WSC

Professional interactions

It's just that I think we need to find time we meet and you know … sit down and iron out some issues and how we should really, you know, kind of interact. [P8, Medical officer]

They should be able to occasionally invite people to go there and see what is it that they do. So that is why they should also … keep in touch. They should continuously inform the incoming officers that there is this department. You guys are functioning in isolation according to me … According to me being me in this hospital you are functioning in isolation … could have informed myself, but how? Actually being part of (department) I could have informed myself when I went there, but then somebody who is not in (department) may not even go there. How do they know about WSC? Why are they not coming forth? [P11, Medical officer]

Experiences as WSC patients

I heard about it when I was suffering, I had ah...I used to have a pain on the… on the lower back. So the doctors used to give me analgesics but with no improvement. So I heard about those people, the spine care program. So I referred myself to... to them. When I got there they said, 'no, go and see the doctor then you should come with an x-ray.' So I went back to OPD (outpatient department). I was seen and I went back to them. So they reviewed my x-ray but there was nothing much abnormal about my x-ray. So they recommended exercises… Eheh… so the first time it was very painful. But after that in the evening you will sleep nicely without any pain. The second visit I went there again. Same problem again. It was painful, the procedure was very painful. They use their elbows on your back like this (demonstrates, with elbow to thigh) and they had some instruments like a… it's a metal rod. Curved. So they are using it like this (motioned with her hand in a rolling motion) ... for the… it's painful. But the effect is very good… after, after that exercise. So I went there about maybe six times. So after that I uh, I discharged myself and the pain there... I can't bear. Last year they phoned me, 'Mma where are you?'. Hey, I am fine now. 'No please come, you have to come and we discharge you.' Eheh... ok I'll come; I haven't been there (laughter). [P14, Nurse]

The first two they went there as walk-ins after hearing of the spine care so they were complaining of the backache so they have since discharged themselves from the therapy on the basis that it is very painful. So it means like the first two, three days they went there they came there with the back being more painful than when they went there. So they decided not to go there. So there is this other one – he went there, he continued with the therapy and I still think even now he is doing the therapy. Yes, at first he was complaining to say it was painful at times also, but now I think he is enjoying. [P4, Allied health]

Role in the healthcare system

It's a very important role they are playing. There are things that we hadn't done – you know it's much more because you're probably sensitizing the community – that's where you start from. So it's much more of the promotive and maybe also preventive aspect there in the community. [P13, Medical officer]

They have to tell the doctors to identify the patients which are … which they can benefit from them, that side. The doctors – they are not – they are far-fetched with information about spine care. That's most of the doctors. Mostly they refer patients for physio even if is a back problem. [P14, Nurse]

Challenges for WSC integration

Referral

So to me there was still no proper channels of referral. Maybe if I see the patients here in (department) and they could benefit from the WSC, that standard of referral is still not yet established. I think that is why maybe you are losing a lot of patients in between. To me, we need to have some standards as to when and how are we going to do those referrals because sometimes when you knock there, there is no one. Sometimes they do come here and they look for someone but there is no one to speak to. So we need a system of referral. [P4, Allied health]

So, I don't know who … whether they are private sector … or they are part of the main hospital setup? But if you are going to – like now, if I want to go there I will not know whether to go through somebody else or go straight to them. [P11, Medical officer]

Sometimes patients present themselves to the WSC and there is a need to refer those patients to the new medical officers or the surgeons but we are still doing it haphazardly. [P4, Allied health]

Organisational culture

You know there is a big problem of knowing some of the professions which were not in the mainstream medical treatment. So you tend to find that even the referring people … people who see patients first … the surgeons – if they are not aware of who is around and what services they provide, they will not [refer]. Some even confuse us because they don't have the in-depth of really what some other professions are doing. For example, there is big confusion between prosthetics, orthotics, occupational therapy, physiotherapy. You find somebody just writes [an unclear referral letter] … he knows something is there but he doesn't know what, how they really … what their parameters are. [P6, Allied health]

Well sometimes I call them, they write letters to us … but I think somehow we need to meet and see how we can work together. [P8, Medical officer]

Our (named) department, most of the time, we are the last people to know about anything that is new in the hospital because from communications that occur in the hospital, you know we are not involved. [P12, Medical officer]

Expansion and improvement

I think the issue of volunteers coming in and going, I think it's going to disturb the program in a way moving forward. Because as Motswana we normally establish that rapport with the people we first see. We need continuity. (long pause) Because as it stands, it's like you are piloting – you want to see whether this thing will run or what. Eventually you won't have that faith and belief in them because we need to see you being established and being yeah, like, well … established and should be having that faith in you. Because you start to ask yourself, what if volunteers are not going to come? Already you have started people with the therapy so what is going to happen to them? [P4, Allied health]
